# Comparative analysis of gene expression profiles in differentiated subcutaneous adipocytes between Jiaxing Black and Large White pigs

**DOI:** 10.1186/s12864-020-07361-9

**Published:** 2021-01-19

**Authors:** Dawei Zhang, Wenjing Wu, Xin Huang, Ke Xu, Cheng Zheng, Jin Zhang

**Affiliations:** 1 College of Biological, Chemical Science and Engineering, Jiaxing University, Jiaxing, 314001 China; 2 College of Agronomy and Biotechnology, Hebei Normal University of Science and Technology, Qin Huangdao, 066000 Hebei China

**Keywords:** Subcutaneous fat, Pig, *Sus scrofa*, LncRNA, mRNA, RNA-seq

## Abstract

**Background:**

Chinese domestic pig breeds are reputed for pork quality, but their low ratio of lean-to-fat carcass weight decreases production efficiency. A better understanding of the genetic regulation network of subcutaneous fat tissue is necessary for the rational selection of Chinese domestic pig breeds. In the present study, subcutaneous adipocytes were isolated from Jiaxing Black pigs a Chinese indigenous pig breed with redundant subcutaneous fat deposition and Large White pigs a lean-type pig breed with relatively low subcutaneous fat deposition. The expression profiles of mRNAs and lncRNAs were compared by RNA-seq analysis to identify biomarkers correlated with the differences of subcutaneous fat deposition between the two breeds.

**Results:**

A total of 1058 differentially expressed genes and 221 differentially expressed lncRNAs were identified in subcutaneous adipocytes between Jiaxing Black and Large White pigs, which included 275 up-regulated mRNAs, 783 down-regulated mRNAs, 118 up-regulated lncRNAs and 103 down-regulated lncRNAs. Gene Ontology and KEGG pathway enrichment analyses revealed that the differentially expressed genes and differentially expressed lncRNAs were mainly involved in the immune response, cell fate determination, PI3K-Akt signaling pathway and MAPK signaling pathway, which are known to be related to adipogenesis and lipid metabolism. The expression levels of differentially expressed genes and differentially expressed lncRNAs according to the RNA-seq data were verified by quantitative PCR, which showed 81.8% consistency. The differences in MAPK pathway activity between Jiaxing Black and Large White pigs was confirmed by western blot analysis, which revealed elevated p38 phosphorylation in Jiaxing Black pigs.

**Conclusions:**

This study offers a detailed characterization of mRNAs and lncRNAs in fat- and lean-type pig breeds. The activity of the MAPK signaling pathway was found to be associated with subcutaneous adipogenesis. These results provide new targets for further investigation of the molecular mechanisms regulating subcutaneous fat deposition in pigs.

**Supplementary Information:**

The online version contains supplementary material available at 10.1186/s12864-020-07361-9.

## Background

Fat deposition is one of the most important economic traits of pigs. The amount of subcutaneous (SC) fat deposits is associated with lean meat carcass percentage, while intramuscular (IM) fat content is the main factor affecting pork quality [1]. Foreign pig breeds, such as Duroc, Large White (LW), and Landrace, deposit low levels of SC fat, while Chinese indigenous pig breeds, such as Laiwu, Taihu, and Jinhua, deposit high levels of SC fat [2–6]. Excessive SC fat deposition greatly decreases the growth performance and meat production efficiency, which results in profit reduction [7, 8]. However, Chinese indigenous pig breeds often exhibit better fertility, disease resistance and IM fat content than foreign pig breeds [9, 10]. Understanding the porcine adipocyte regulation network to decrease SC fat deposition is a key issue in genetic improvement of Chinese indigenous pig breeds. In addition, human health problems caused by excessive fat accumulation are becoming increasingly common. It has been demonstrated that obesity increases the risk for the development of type 2 diabetes mellitus, cardiovascular disease, hypertension, dyslipidemia, and certain types of cancer [11, 12]. Notably, pigs can be used in biomedical studies due to their anatomic and physiological similarity to humans [13, 14]. Therefore, clarifying the molecular mechanisms of SC fat deposition in pigs can not only benefit the genetic breeding of pigs, but also deepen our understanding of human metabolic diseases.

Porcine SC fat deposition is largely determined by the proliferation and differentiation of adipocytes [15]. With the advent of omics technologies, many genes and pathways regulating the metabolism of porcine adipocytes have been identified [16–18]. Zhang et al. [19, 20] reported that apolipoprotein R is the key molecule promoting lipolysis in porcine adipocytes according to DNA microarray analysis. Wu et al. [21] demonstrated that C1q/tumor necrosis factor-related protein 6 (*C1QTNF6*) regulates porcine SC fat deposition via the MAPK and p53 signaling pathways using RNA-seq analysis. Recently, the regulatory role of long noncoding RNAs (lncRNAs) in porcine adipogenesis has garnered increasing attention [22, 23]. LncRNAs are defined as a class of transcribed RNA molecules that are more than 200 nucleotides in length and do not encode proteins [24]. LncRNAs can interact with DNA, RNA or proteins, and regulate gene expression via diverse mechanisms [25]. Identifying the regulatory role of lncRNAs in porcine adipogenesis is of great importance for understanding the molecular mechanisms that regulate SC fat deposition in pigs. Although several reports on lncRNAs in porcine adipose tissue were published in the past 2 years, our understanding how lncRNAs regulate fat deposition in pigs is still very limited.

Jiaxing Black (JX) pig, a Chinese indigenous pig breed in the Taihu Lake region, is characterized by its early sexual maturity, high fecundity and crude feed tolerance. Additionally, it is renowned for the good performance of its hybrids with foreign pig breeds, and plump muscles with a high content of IM fat. Products derived from JX pigs have been developed into a well-recognized commercial pork brand in China [26]. However, the excessive SC fat deposition decreases the growth efficiency and results in profit reduction. By contrast, LW pigs are the most widely distributed lean-type pig breed with relatively low SC fat deposition [10]. In this study, high-throughput RNA-seq was conducted to compare the gene expression profiles of differentiated SC adipocytes from the two pig breeds. LncRNAs and genes associated with porcine adipogenesis or lipid metabolism were identified. Furthermore, functional enrichments and interaction network analyses were conducted to investigate the molecular mechanisms of differentially expressed lncRNAs (DELs) and genes regulating fat deposition, which provides new relevant data for understanding the regulatory network of SC fat deposition in pigs.

## Results

### RNA-seq analysis of SC adipocytes from JX pig and LW pig

Primary SC adipocytes were isolated from three JX and three LW pigs (3 days old), and subjected to 8 days of differentiation. The differentiated adipocytes were harvested and subjected to RNA-seq analysis in three biological replicates. The Illumina HiSeq 2000 platform provided an average of 15.2GB of clean reads for each sample. The percentage of clean reads among the raw data in each library ranged from 91.44 to 95.10%. For each sample, 90.22, 87.20, 86.05, 84.79, 85.59 and 85.76% were uniquely mapped to the current version of the pig genome (Sscrofa 11.1), representing 12,446, 11,643, 11,765, 11,579, 11,593 and 11,310 genes, respectively (Additional file 1: Supplementary Table 1). Gene numbers within a defined range of FPKM values (FPKM ≤1, FPKM 1 ~ 10 and FPKM ≥10) were analyzed, and each sample gave similar results (Additional file 1: Supplementary Fig. 1A). In each breed, the abundance of mRNAs was relatively higher than that of lncRNAs, as expected, while both mRNAs and lncRNAs showed similar distribution in both breeds (Additional file 1: Supplementary Figs. 1B and 1C). The transcripts that met at least three of four criteria (CPC, txCdsPredict, CNCI and Pfam) were identified as candidates lncRNAs, which yielded 4165 lncRNAs for subsequent analysis (Additional file 1: Supplementary Fig. 1D). The majority of known lncRNAs have two to four exons, while the novel lncRNAs mainly had one to three exons (Additional file 1: Supplementary Fig. 1E). In addition, the transcript abundance of housekeeping genes such as *EEF1A1*, *ACTA2* and *GAPDH* was high, as can be seen in supplementary Table 2 (Additional file 1). Taken together, both the biological replicates and sequencing data indicated sufficiently good data quality for further analysis.

### Differentially expressed lncRNAs and genes in SC adipocytes from the two breeds

**Fig. 1:**
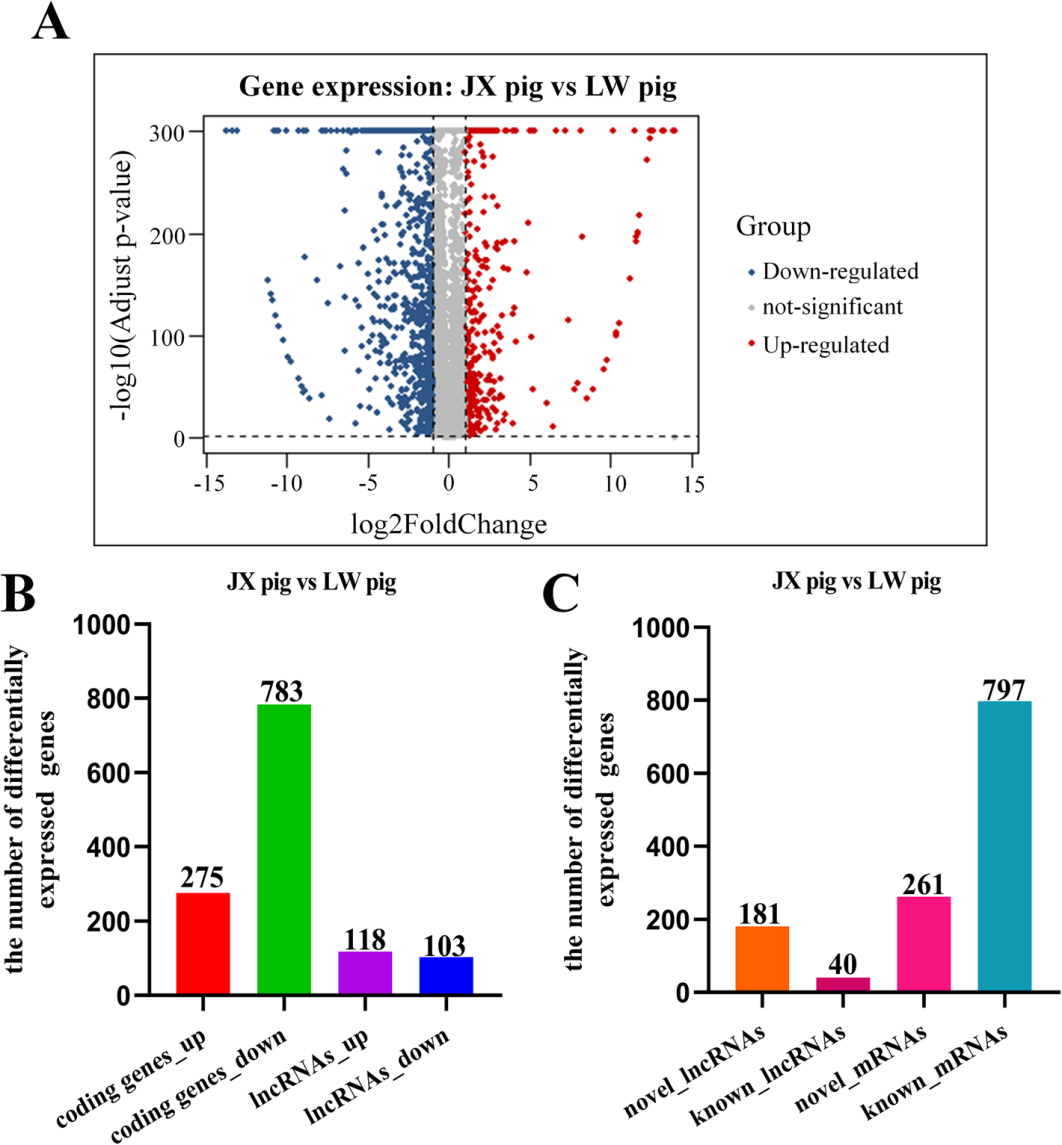
Differential expression characteristics of coding genes and lncRNAs in SC adipocytes between LW and JX pigs. **a** The volcano plot of differentially expressed coding genes and lncRNAs. **b** Quantitive comparison of the up- and down-regulated genes. **c** The number of known and novel genes

To further understand the differences of SC adipocytes between the two breeds, comparative transcriptome analysis was conducted, and the minimum FPKM value of gene expression was greater than or equal to 1. A total of 1279 genes (1058 coding genes and 221 lncRNAs) were differentially expressed, including 393 up- and 886 down-regulated genes in SC adipocytes between the two breeds (Fig. 1a). Among the 1058 differentially expressed genes (DEGs), 275 were up- and 783 were down-regulated, and the FPKM values of some DEGs exhibited great differences between JX and LW pigs, including *KRT5*, *UBC*, *LDHB*, *C1QTNF3* and *RAMP1* (Additional file 1: Supplementary Table 3). Among the 221 DELs, 118 were up- and 103 were down-regulated (Fig. 1b). Among these DEGs and DELs, 797 DEGs and 40 DELs had been previously annotated, and 261 DEGs and 181 DELs were novel (Fig. 1c).

### Functional enrichment analysis of DEGs

**Fig. 2:**
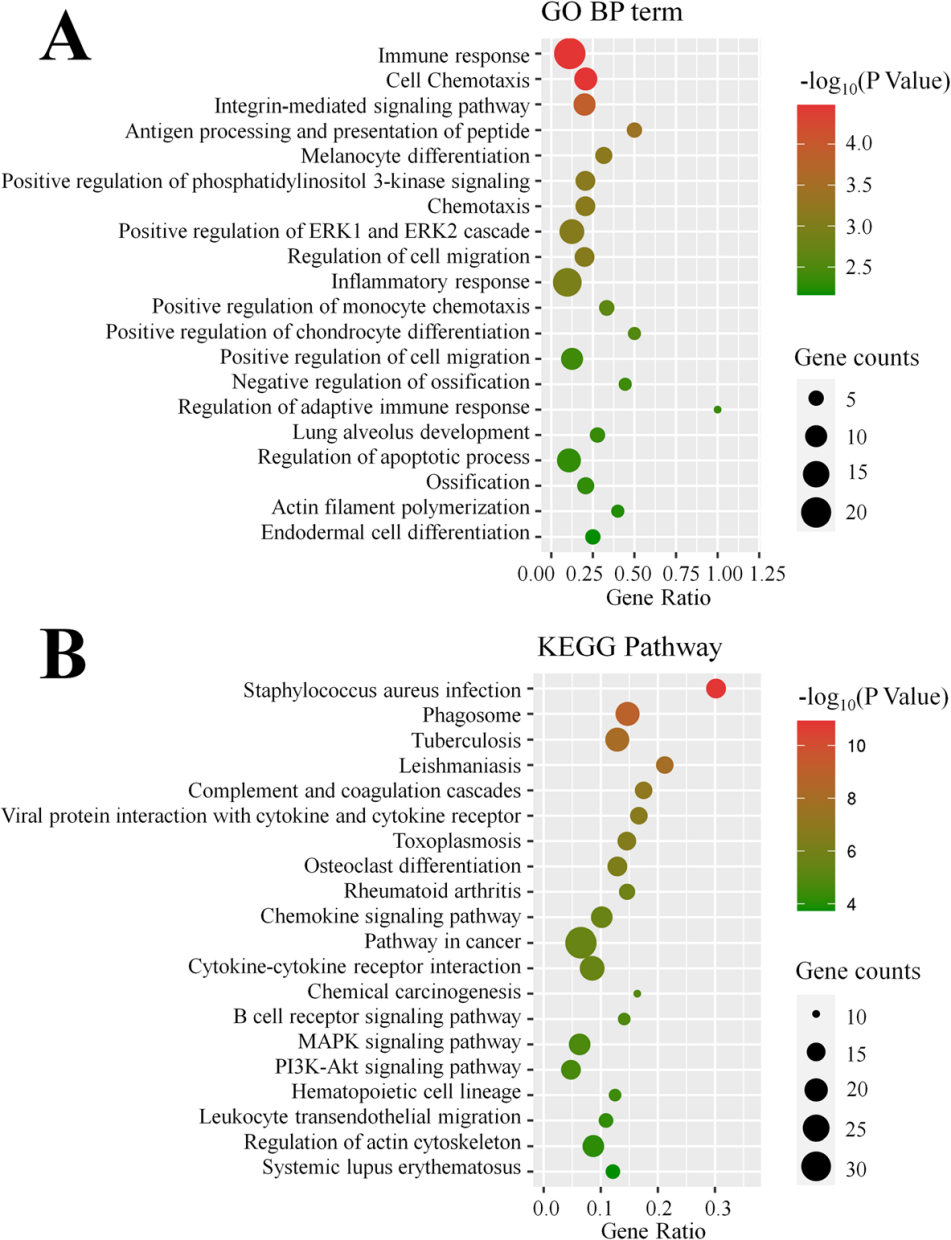
GO annotation and KEGG pathway analysis of DEGs. **a** Go terms distribution of DEGs under biological processes. **b** Enrichment of DEGs in signaling pathways. Each bubble represents a term. The size of the bubble indicates the number of involved genes. The colors indicate *P* values, and the significance level of enrichment was set at *P* < 0.05, and enrichment terms was ranked by *P* values

The potential functions and signaling pathways of all DEGs were subjected to Gene Ontology (GO) and Kyoto Encyclopedia of Genes and Genomes (KEGG) enrichment analysis. GO analysis based on biological process was conducted and the top 20 most highly enriched categories with *P* < 0.05 were listed (Fig. 2a). The results showed that DEGs related to cell differentiation, migration, and apoptosis were significantly enriched. Five processes related to immunity, namely “Immune response”, “Antigen processing and presentation of peptide”, “Inflammatory response”, “Positive regulation of monocyte chemotaxis”, and “Regulation of adaptive immune response”, were detected. Two terms closely associated with lipid metabolism were also identified, including “Positive regulation of phosphatidylinositol 3-kinase signaling” and “Positive regulation of ERK1 and ERK2 cascade”. In addition, KEGG enrichment analysis was performed and the top 20 pathways are presented in Fig. 2b. Among these results, several immune-related pathways were also found, such as “*Staphylococcus aureus* infection”, “Phagosome”, “Tuberculosis”, “Complement and coagulation cascades”, “Viral protein interaction with cytokine and cytokine receptor”, “Rheumatoid arthritis”, “Chemokine signaling pathway”, “Cytokine-cytokine receptor interaction”, “B cell receptor signaling pathway”, “Leukocyte transendothelial migration”, and “Systemic lupus erythematosus”. Moreover, “Osteoclast differentiation”, “MAPK signaling pathway” and “PI3K-Akt signaling pathway” were significantly enriched, all of which are highly associated with adipocyte differentiation and lipid accumulation. The results of enrichment analysis illustrated the regulatory differences of SC fat deposition between JX and LW pigs.

### Protein-protein interaction network analysis

**Fig. 3:**
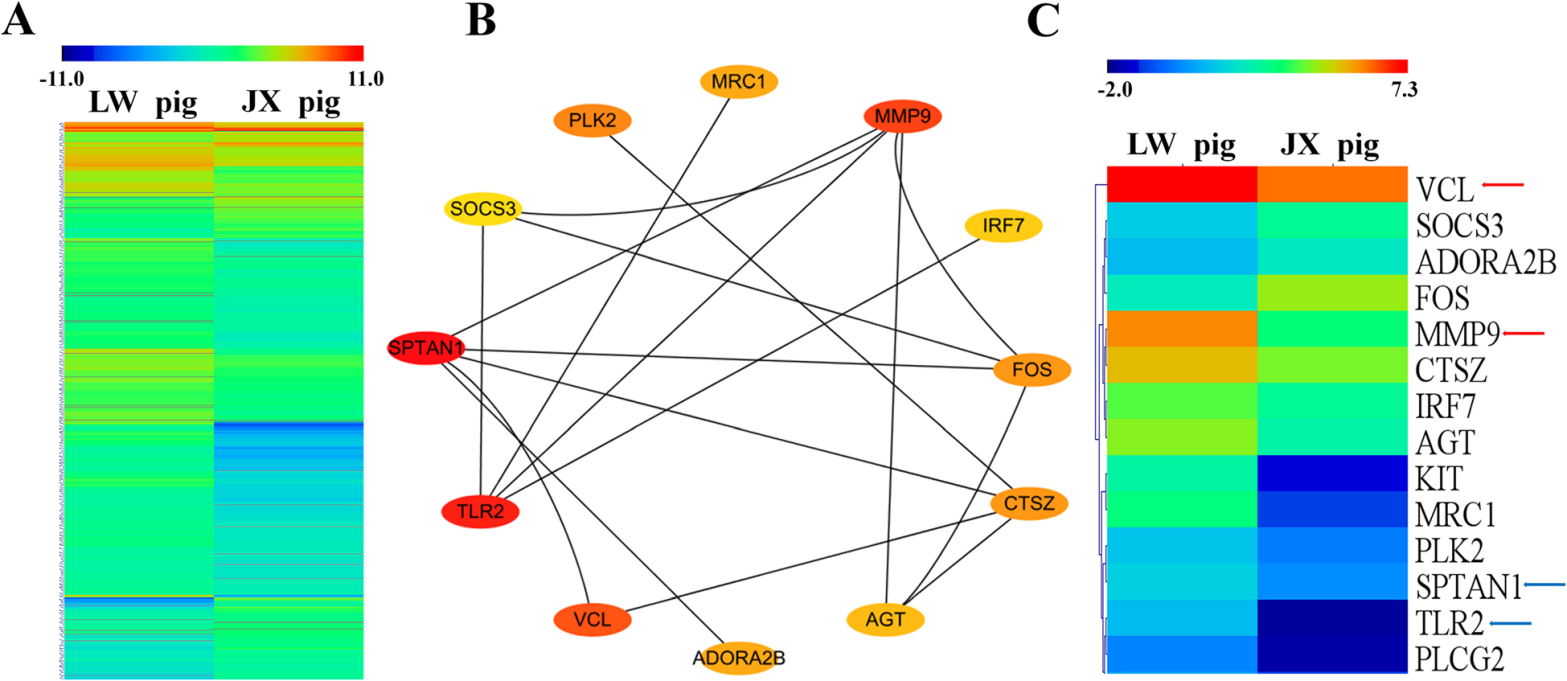
Protein-protein interaction (PPI) network analysis by cytoscape of DEGs. **a** Unsupervised hierarchical clustering of the expression profile of significant genes of DEGs. **b** The PPI network of DEGs according to MCODE. **c** Unsupervised hierarchical clustering of the expression profile of hub genes in PPI network, and the arrows (red: up-regulated; blue: down-regulated) indicate four DEGs with high interaction scores

In unsupervised hierarchical clustering analysis, heat maps were generated using the DEGs, and they clearly self-segregated into different clusters for JX and LW pigs. These results reflected the distinct mRNA expression profiles of the two breeds (Fig. 3a). The protein-protein network was constructed based on the Maximal Clique Centrality (MCC) topological algorithm. According to the interaction scores, four DEGs exhibited obvious strong connections with other genes. Among the four DEGs, *MMP9* and *VCL* were up-regulated, while *SPTAN1* and *TLR2* were down-regulated in JX pigs compared to LW pigs (Fig. 3c).

### Functional enrichment analysis of lncRNAs based on target genes

**Fig. 4:**
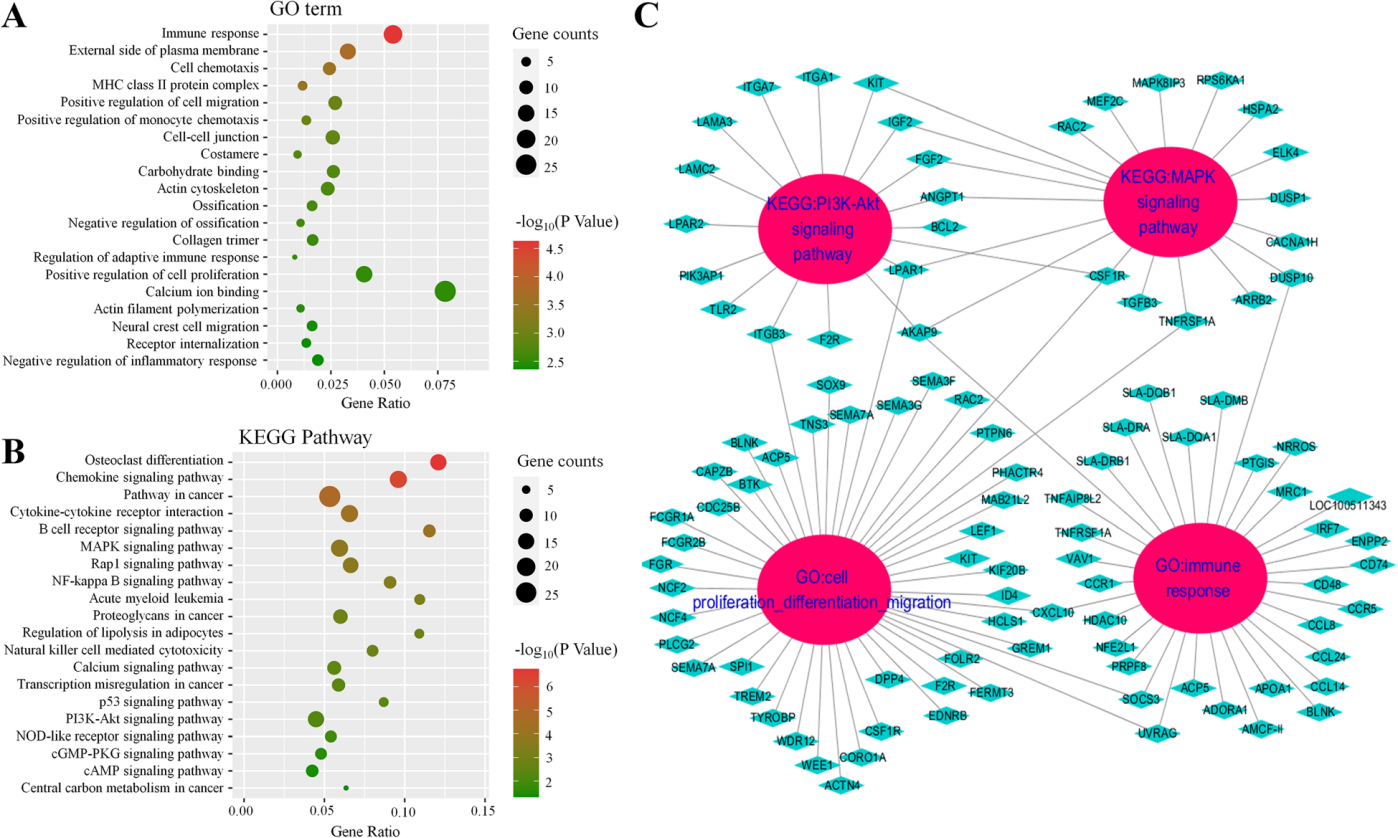
Functional enrichment and PPI network analysis of DELs-target genes. GO enrichment (**a**) and KEGG pathway enrichment (**b**) of the target genes. **c** Enrichment network of target genes of DELs

Based on the RNA-seq data, the potential target genes of DELs were predicted to explore their potential functions (Additional file 2: Supplementary Table 4). In GO enrichment analysis, the five immunity-related terms “Immune response”, “MHC class II protein complex”, “Positive regulation of monocyte chemotaxis”, “Regulation of adaptive immune response” and “Negative regulation of inflammatory response”, were detected. The categories cell chemotaxis, cell migration and ossification, which were detected in the DEG analysis, were also identified here (Fig. 4a). In KEGG enrichment analysis, seven pathways related to adipocyte differentiation and lipid accumulation were identified, including “MAPK signaling pathway”, “Regulation of lipolysis in adipocyte”, “Calcium signaling pathway”, “p53 signaling pathway”, “PI3K-Akt signaling pathway”, “cGMP-PKG signaling pathway”, and “cAMP signaling pathway” (Fig. 4b). In addition, the target genes enriched in four categories, including “MAPK signaling pathway”, “PI3K-Akt signaling pathway”, “Immune response”, and “Cell proliferation, differentiation and migration” were shown in Fig. 4c, and they were also identified in the enrichment analysis of DEGs. Among the target genes, *WDR12, LPAR1, WEE1, CDC25B, CAPZB* and *UVRAG* are known to participate in the regulation of cell proliferation or differentiation. Furthermore, *PRPF8, AKAP9, UVRAG, HDAC10* and *NFE2L1* are known to mediate the immune response. Moreover, *LPAR1* and *AKAP9* were also reported to be associated with the PI3K/AKT signaling pathway or MAPK signaling pathway. Significantly, *Nfe2l1* was reported to have an impact on the plasticity of adipose tissue. Thus, the DELs might play an essential role in the distinct adipogenesis of JX pigs.

### Validation of the DEGs and DELs

**Fig. 5:**
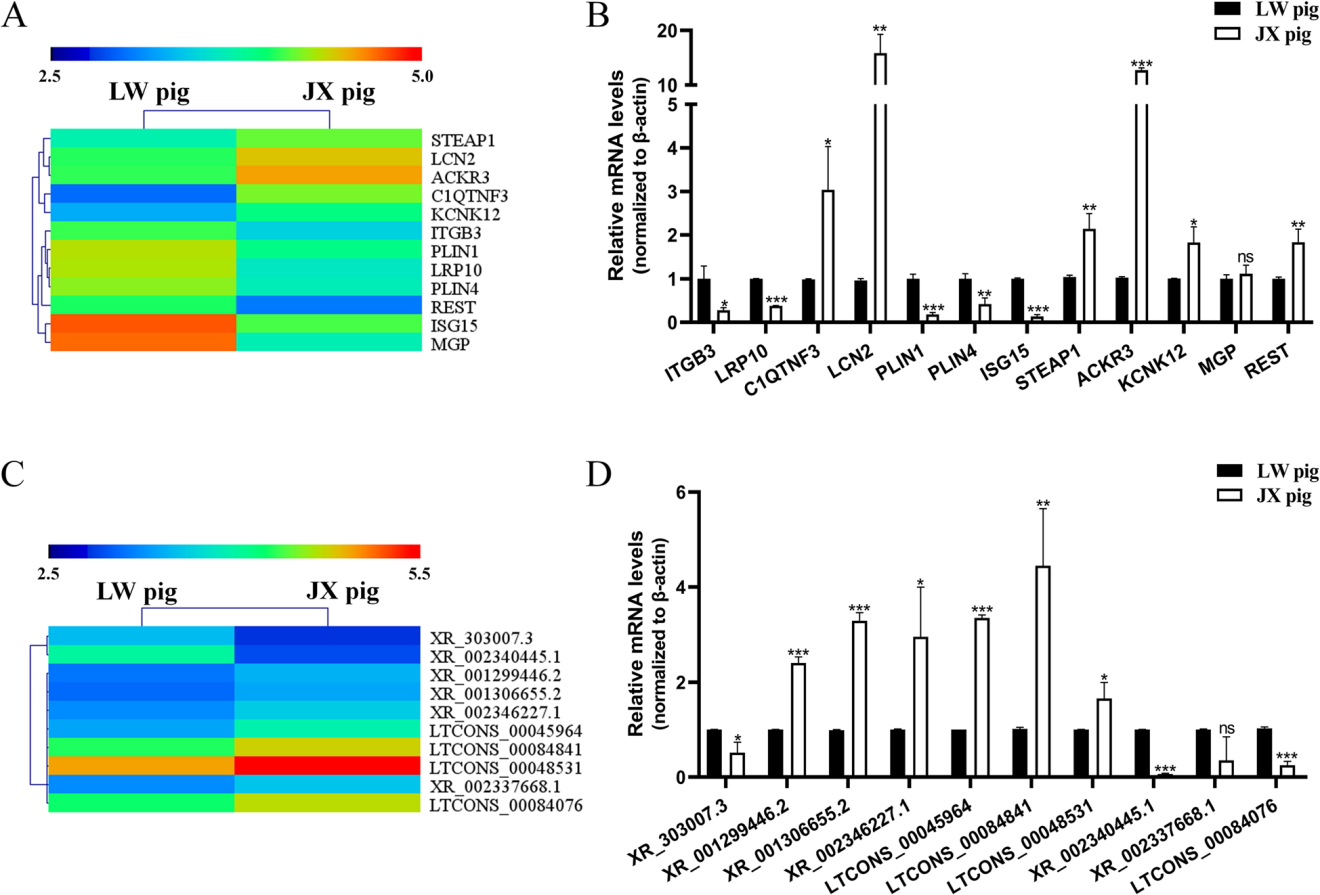
Q-PCR validation of DEGs and DELs in differentiated SC adipocytes between LW and JX pigs. **a** Unsupervised hierarchical clustering of the expression profile of twelve randomly selected DEGs. **b** Q-PCR validation of the expression level of twelve randomly selected DEGs. **c** Unsupervised hierarchical clustering of the expression profile of ten randomly selected DELs. **d** Q-PCR validation of the expression level of ten randomly selected DELs. *: *P* < 0.05; **: *P* < 0.01; ***: *P* < 0.001

To validate the reliability of the RNA-seq results, 12 DEGs and 10 DELs were randomly chosen for quantitative PCR (qPCR) verification (Fig. 5a-d). Compared with the RNA-seq data, 10 DEGs and 8 DELs gave consistent results, while two DEGs (*MGP* and *RESTI)* and two DELs (XR_002337668.1 and LTCONS_00084076) showed statistically different results by qPCR analysis. Overall, 81.8% of the results were in agreement between the two techniques.

### Verification of the pathway analysis

**Fig. 6:**
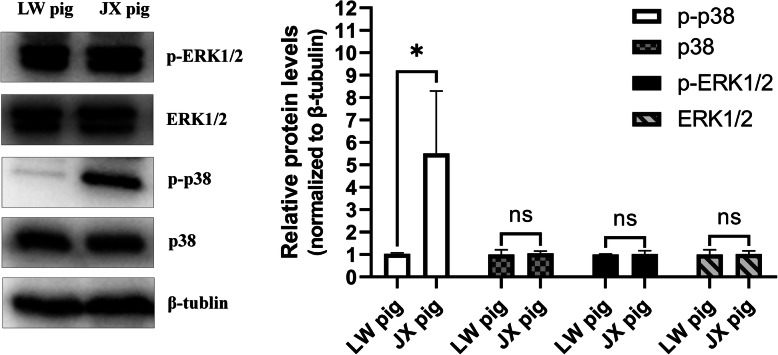
Verification of MAPK signaling pathway by western blot. The total protein abundance and phosphorylated level of p38 and ERK1/2 were observed in SC fat tissue of LW and JX pigs, and the protein level of β-tubulin was used as a control. Quantification of the protein levels is shown on the right, and the results are expressed as the means ± SE of three samples. *: *P* < 0.05; ns: not significant

Because the MAPK signaling pathway was identified in the functional enrichment analysis of both DEGs and DELs, its activity in SC fat tissues of the two breeds was examined. Expression levels and phosphorylation of two kinases in the MAPK pathway, ERK1/2 and p38, were determined by western blot analysis. ERK1/2 showed no differences in total protein abundance or phosphorylation between the two breeds. However, while the total protein abundance of p38 was similar, the abundance of phosphorylated p38 showed an obvious difference between the two breeds. Accordingly, the samples from JX pigs showed higher p38 phosphorylation levels than the samples from LW pigs (Fig. 6 and Additional file 3: Supplementary Fig. 2). The difference of p38 phosphorylation between the two breeds supported the results of pathway enrichment analysis.

## Discussion

SC fat tissue has multiple functions in pigs, including thermal insulation, energy storage and adipokine secretion [27, 28]. However, the reduction of SC fat content is of great importance for pig production because fat deposition wastes a lot of energy [7]. Therefore, excessive triglyceride accumulation in SC fat tissues is unfavorable for both energy utilization and lean meat production in pigs. Many Chinese domestic pig breeds are reputed for their high pork quality, but their low ratio of lean-to-fat carcass weight decreases production efficiency. Consequently, a better understanding of the regulation network controlling SC fat tissue deposition is necessary for the rational genetic improvement of Chinese domestic pig breeds. In this study, the gene expression profiles of SC adipocytes from the local JX pig breed and foreign LW pig breed were compared via RNA-seq analysis. A total of 1279 differentially expressed genes were identified, including 1058 coding genes (DEGs) and 221 lncRNAs (DELs). These results were validated by qPCR analysis, which indicated that the data are reliable, with 81.8% consistency. Interestingly, we noticed that there were more down-regulated DEGs than up-regulated DEGs. This result indicated that many genes are suppressed in the SC adipocytes of JX pigs, which may be related to vigorous adipogenesis inside the cells.

In order to identify the differences between the regulation networks of SC adipocytes from the two pig breeds, GO and KEGG pathway enrichment analyses were performed. DEGs and target genes of DELs were mainly enriched in three pathways related to lipid metabolism and adipocyte differentiation, namely “Calcium signaling pathway”, “PI3K-Akt signaling pathway” and “MAPK signaling pathway”. The calcium signaling pathway can regulate lipolysis and the accumulation of adipose tissue by changing the concentration of calcium ions in adipocytes [29, 30]. PI3Ks are a group of intracellular lipid kinases, which phosphorylate phosphatidylinositol and phosphoinositide to generate new intracellular second messengers [31]. These messengers in turn activate many intracellular signaling pathways and regulate various biological process in cells [32]. Plum et al.[33] reported that the PI3K signaling pathway is induced by leptin and participates in biological processes related to obesity. Insulin signaling via the PI3K/Akt axis plays an important role within adipocytes of obese patients, where the excess of lipids has to be properly stored in fat tissue [34]. The MAPK signaling pathway is common to several cell types, and it is involved in many biological processes, including cell proliferation, differentiation, development, and apoptosis [35]. The MAPK signaling pathway was among the top 20 most highly enriched pathways in KEGG analysis, and the GO annotation of DEGs uncovered the ERK1-ERK2 cascade, which indicates the activity of MAPK signaling pathway [36]. Several studies have shown that the MAPK pathway regulates the expression of adipogenic transcription factors during the adipogenesis [37]. The oncogenic form of Ras protein is a strong activator of the ERK pathway, and transfection of 3 T3-L1 preadipocyte cells with an activated Ras expression vector led to growth arrest and terminal adipocyte differentiation. The expression of the key adipogenic regulators C/EBPα, β, and δ, as well as PPARγ requires ERK activity, but the phosphorylation of PPARγ caused by ERK inhibits adipocyte differentiation [38]. A study of the role of p38 MAPK signaling in regulating fat deposition in mice suggested that p38 MAPK could inhibit adipogenic differentiation by inhibiting the activity and expression of C/EBPβ and PPARγ during the whole process of lipogenesis in mice [39]. Owing to the opposing roles described for the MAPK pathway, one hypothesis states that the activation of MAPK can have different effects depending on the stage of differentiation. This hypothesis is in agreement with the fact that the differentiation process of 3 T3-L1 adipocytes requires a specific proliferative step (called mitotic clonal expansion, MCE) at the beginning [38, 40]. In addition, p38 MAPK activity was significantly higher in preadipocytes than in adipocytes [39], again suggesting that p38 MAPK activity has to be regulated in a timely manner during adipocyte differentiation. Therefore, the results of this study indicate that the MAPK signaling pathway may be important for mediating SC adipogenesis in JX pigs, and the difference between the two breeds may be related to mitogenic activity in preadipocytes regulated by the MAPK pathway. Considering that ERK and p38 are two main kinases in the MAPK pathway, and opposite results were observed for the regulatory functions of ERK and p38 during adipocyte differentiation [38, 41, 42], we further compared the activation of p38/ERK in SC adipose tissue of the two pig breeds. JX pigs showed a higher level of p38 phosphorylation in adipocytes than LW pigs. Previous research showed that the p38 MAPK signal could negatively regulate preadipocyte differentiation as well as SC and IM fat deposition in broiler chickens [43], as well as inhibit lipogenesis in 3T3-L1 adipocyte [44]. However, most studies performed in cell lines described a positive role of p38 in adipogenesis, and a decrease of C/EBPβ phosphorylation as well as PPARγ transactivation activity was observed when p38 activation was inhibited [38]. Moreover, mice lacking p38α in adipose tissues displayed a lean phenotype, improved metabolism, and resistance to diet-induced obesity[45]. Thus, a precise understanding of the regulatory mechanism of these two pathways will be crucial for revealing the reason why JX pigs have a lower lean carcass weight ratio, and will be helpful for efficiently treating obesity.

Healthy adipose tissue is minimally infiltrated by immune cells, which serve as sentinels to detect invaders [46, 47]. These cells also exert housekeeping functions that help maintain tissue integrity [48, 49]. In this study, we discovered that many immune-related signaling pathways, such as the inflammatory response, adaptive immune response, tuberculosis, B cell receptor signaling pathway, phagosome and cytokine-cytokine receptor interaction, were enriched in GO and KEGG enrichment analysis. Thus, the obtained differential expression profiles of mRNAs and lncRNAs indicated that there are potential functions of cytokines and immune cells in the fat deposition and metabolism of pigs. Previous reports found that adipose tissue contains not only adipocytes but also fibroblasts, endothelial cells and a wide array of immune cells (adipose tissue macrophages, neutrophils, mast cells, eosinophils, T and B cells) that maintain tissue homeostasis [50]. The adipocyte expansion caused by a positive energy balance results in the expression of chemoattractant molecules and infiltration of inflammatory cells [51]. This difference of immune-related signaling pathways may be associated with the excessive SC fat deposition in JX pigs. However, the adipose tissue-derived adipokines are also crucial players in inflammation and immunity. For example, leptin has been shown to regulate both innate and adaptive immune responses, and its receptor is expressed throughout the immune system [52]. Proinflammatory cytokines, which promote lipolysis, have been traditionally considered to antagonize adipocyte differentiation. Nevertheless, transient low-level inflammation actually stimulates adipogenesis in animal models, and a transient inflammatory response is essential for healthy expansion of white adipose tissue through adipocyte progenitor recruitment [53]. Thus, there’s a link between the immune response and fat metabolism, and it controls the homeostasis of adipose tissue. In addition to maintaining the structural integrity, these pathways can also regulate the endocrine functions of adipose tissues [54, 55]. Consequently, our results provide evidence that SC adipogenesis in pigs is related to the immune landscape of porcine adipose tissues.

In the protein-protein interaction network of the identified DEGs, twelve hub genes were identified by the MCC topological algorithm. *VCL* encodes the cytoskeletal focal adhesion protein vinculin, and its depletion in mesenchymal stem cells (MSCs) promoted adipocyte differentiation on rigid substrates, as well as markedly increasing the mRNA expression of adipogenesis markers *PPARγ2* and *aP2* [56]. It has been reported that various TLRs, such as NOD1, and NOD2, are expressed in adipocytes and adipose tissue of mouse and human origin. Moreover, the proinflammatory environment induced by the activation of TLR4 or TLR2 leads to the suppression of adipocyte differentiation [57]. Interferon regulatory factor 7 (IRF7) physically interacts with GSDMD and subsequently forms a complex to promote adipocyte pyroptosis [58]. The lysosomal protease cathepsin Z (*CTSZ*) is necessary for mitochondrial respiration in both mouse and human brown adipocytes, and it is markedly induced by cold [59]. In our study, a lower expression of these genes in JX pigs than in LW pigs was observed. *SOCS3* has been reported to play a central role in metabolism as well as regulate the expression of *SREBP1c*, which is an important transcriptional factor of genes involved in lipid synthesis. Furthermore, *SOCS3* gene variations are associated with disturbances of lipid metabolism [60]. The transcription factor FOS is a member of the AP-1 complex, and knockdown of *FOS* abolished the ongoing differentiation process [61]. Adipocyte-derived angiotensinogen (*AGT*) plays a role in both local adipose tissue development and in the endocrine system, and mice that overexpress *AGT* in adipose tissue developed obesity with adipocyte hypertrophy, concurrent with insulin resistance and increased expression of lipogenic and pro-inflammatory makers [62]. It should be noted that other types of proteinases are also known to be involved in adipogenesis. Some matrix metalloproteases (MMPs) are expressed in adipose tissues, where they can act as a paracrine factor as well as secreted into the blood. Several groups showed that MMP2 and MMP9 possess adipogenesis-enhancing activity [63]. In the predicted protein-protein interaction network constructed in this study, *MMP9* is located at a key central node. Our results show that *SOCS3*, *FOS*, *AGT* and *MMP9* have higher expression in JX pigs. Taken together, these regulatory relationships may partly explain the mechanism of porcine SC deposition.

The biological function of lncRNAs is usually mediated by their target genes. Thus, the target genes of the identified DELs were predicted and subjected to enrichment analysis. Our results indicated that the lncRNAs LTCONS_00049888, LTCONS_00077321, LTCONS_00060219, LTCONS_00006788, LTCONS_00055593, LTCONS_00029009, LTCONS_00031822 and XR_002341580.1 regulate *AKAP9*, *CDC25B*, *LPAR1*, *Nfe2l1*, *UVRAG*, *CAPZB* and *WEE1*, respectively. *AKAP9* regulates activation-induced retention of T lymphocytes at sites of inflammation and is related to the activation of the MAPK pathway in thyroid cancer [64]. In addition, *CDC25B*, *LPAR1*, *UVRAG* and *CAPZB* regulate cell growth, morphology and differentiation. It is worth noting that LTCONS_00055593 may play critical roles in fat metabolism and deposition by regulating its target gene *Nfe2l1*, which affects the plasticity of adipose tissue [65]. These results indicate that the DELs might participate in cell proliferation or differentiation, and their function in SC adipogenesis should be confirmed by further analysis in future studies.

The DEGs and DELs identified in this study possibly reflect differences of fat deposition or deeper genetic differences between the two breeds. According to GO and KEGG analysis, several pathways related to lipid metabolism were enriched, including“positive regulation of ERK1 and ERK2 cascade”, “Calcium signaling pathway”, “PI3K-Akt signaling pathway”, “Osteoclast differentiation”, and “MAPK signaling pathway”, which may account for the difference of fat deposition between the two breeds. Western blot analysis confirmed the results of enrichment. Our findings expand the knowledge of the regulatory network in porcine SC adipocytes, which is necessary for the rational breeding of pigs for improved fat-related traits.

## Conclusions

In summary, a comparative transcriptome analysis of porcine SC adipocytes between JX and LW pigs was conducted. A large number of DEGs and DELs were identified. Elevated activity of the MAPK/p38 pathway was detected in the SC fat of JX pigs. Taken together, the results may help explain the excessive fat deposition of JX pigs and offer a clue for genetic improvement of Chinese domestic pig breeds.

## Methods

### Experimental animals

All experimental procedures involving animals were performed in accordance with the guidelines of the Animal Care and Use Committee at the Jiaxing University. The experimental animals used here included 3 male JX pigs and 3 male LW pigs, which were 3-day-old and provided by Zhejiang Qinglian Food Co., Ltd. (Jiaxing, Zhejiang Province, China). The piglets were raised under the same feeding and environmental conditions. All piglets were sacrificed using a CO_2_ euthanasia box, after which the SC adipose tissues were collected for the primary culture of SC adipocytes.

### Preadipocyte culture and differentiation

Porcine SC adipose tissue isolated from male piglet was washed three times with serum-free DMEM/F12 medium. Then, tissues were aseptically cut into pieces and incubated with 1 mg/mL type I collagenase (Invitrogen, Carlsbad, CA, USA) at 37 °C for an hour. The digestion solution was filtrated through a 200 μm nylon mesh, after which the preadipocytes were collected by centrifuged at 1000 rpm for 10 min, and cultured in DMEM/F12 medium (HyClone, USA) containing 10% fetal bovine serum (FBS; Gibco, USB) and 1% penicillin-streptomycin at 37 °C in a humidified atmosphere comprising 5% CO_2_. After the preadipocytes reached confluence (designated as experimental day 0), the differentiation cocktail comprising DMEM/F12 supplemented with 10% FBS, 0.5 mM isobutyl methylxanthine (IBMX; Sigma, USA), 0.5 mM dexamethasone (Sigma, USA), and 20 nM insulin (Sigma, USA) was added to induce cell differentiation for 2 days, after which the cells were maintained in DMEM/F12 medium supplemented with 10% FBS and 20 nM insulin for an additional 8 days.

### RNA isolations and Illumina sequencing

Differentiated SC adipocytes from three biological replicates were subjected for RNA isolation using Trizol reagent (Invitrogen, USA). The RNA quality and concentration were measured using a NanoDrop One (Thermo Fisher Scientific, USA) and agarose gel electrophoresis. The RNA was stored at − 80 °C until further use. Ribosomal RNA from each sample was removed using the Ribo-Zero™ rRNA Removal Kit (Epicentre, USA), after which the purified RNA was fragmented and reversely transcribed to synthesize the first-strand cDNA using the TruSeq® First-Strand kit (Illumina, USA). The double-stranded cDNA (ds-cDNA) was synthesized in a reaction mixture comprising buffer, dNTPs, RNase H and DNA polymerase I, and the end of ds-cDNA was ligated with an ‘A’ base and sequencing linker. The entire library was completed by performing amplification of ligation products, and the paired-end sequencing reads generated by the Illumina 2000 platform had a length of 2 × 90 bp.

### Reference genome mapping and gene quantification

After removing low-quality reads from the raw reads using SOAP, the remaining sequences were aligned to the reference genome Sscrofa 11.1 using HISAT software. The resulting alignment data were then fed into StringTie for transcriptome assembly, and Cufflinks was used to map the sequencing transcripts to reference transcripts. Subsequently, the abundance of transcripts was determined using the Fragments Per Kilobase of exon per Million fragments mapped (FPKM) method.

### LncRNA identification

The criteria for candidate lncRNAs identification were as follows: Firstly, transcripts shorter than 200 nt or less than two exons were filtered out; Secondly, the tools encoding potential calculator (CPC), coding-non-coding index (CNCI), txCdsPredict and protein folding domain database (Pfam) were used for lncRNA screening. The lncRNAs that appeared as hits in at least three of the four software tools were included in the final result. To identify the known lncRNAs, lncRNA candidates were aligned to the ALDB database (A Domestic Animal Long Noncoding RNA Database) with the following settings: identity (100%), mismatch (0), E-value (<1e-10) and gap opening (0).

### Differential expression analysis

The RSEM [66] software package was used to quantify gene abundance, and StringTie [67] was used to calculate FPKMs of both lncRNAs and coding genes in each sample. The differential expression analysis of genes with average FPKM of ≥1.0 in a pig breed was performed using the DESeq2 R package [68]. Genes and lncRNAs with an adjusted *P* value < 0.05 and absolute fold change ≥2 were considered to be differentially expressed. A volcano plot of differentially expressed genes was rendered using the ggpubr R package, and heat maps were plotted using MeV 4.9 software.

### LncRNA target gene prediction

LncRNAs can regulate target genes by acting in cis or in trans, and genes with similar expression patterns might exhibit high correlation in biological functions. Accordingly, the target genes of lncRNAs were predicted as follows. For the significance of the proximity of coregulated lncRNA and mRNAs, Spearman and Pearson correlation coefficients were calculated based on the expression values of each lncRNA and mRNA, and co-expressed lncRNA-mRNA pairs with correlation coefficients ≥0.6 were selected. When these target genes were located 10 kb upstream or 20 kb downstream of the lncRNAs, they were considered as cis target genes. Beyond this location, RNAplex [69] was applied to select trans-acting target genes according to the interaction between lncRNA and mRNA sequences with binding energy < − 30.

### Enrichment analyses and construction of the protein-protein interaction network

GO annotation and KEGG pathway enrichment analysis were performed for DEGs and lncRNA target genes to explore the main biological functions of the differentially expressed mRNA and lncRNAs. The statistical enrichment of DEGs was analyzed using DAVID 6.8. The STRING website was used to construct a protein-protein interaction network, and the Cytoscape software was used to visualize it.

### Quantitative real-time RT-PCR

Gene and lncRNA specific primers were designed using the Primer-BLAST tool, and the amplification efficiency of primers was confirmed by general PCR (Additional file 1: Supplementary Table 5). Approximately 0.5 μg of each RNA sample was used to synthesize cDNA templates using the HiFiScript gDNA Removal cDNA kit (CWbiotech, China). A QuantStudio3 Real-time PCR Instrument (Thermo Fisher Scientific, USA) was used for the qRT-PCR assay, and the reaction system was set up according to the manufacturer’s instructions of the 2 × Plus SYBR real-time PCR kit (CWbiotech). The temperature program encompassed an initial denaturation step at 94 °C for 3 min, followed by 40 cycles of 95 °C for 15 s, 60 °C for 15 s, and 72 °C for 30s. The housekeeping gene *β-actin* was used as the control for normalization, and the experiments were performed in triplicate. The 2^-ΔΔCT^ method was used to calculate the relative gene expression levels.

### Western blot analysis

RIPA buffer (Beyotime, Shanghai, China) supplemented with protease inhibitor (Pierce, Bradenton, Florida, USA) was used to extract the total proteins from SC fat tissue of three JX and three LW pigs (3 days old). The lysates were centrifuged at 12000 rpm for 10 min, and the supernatant was boiled in sodium dodecyl sulfate (SDS) loading buffer (Beyotime, Shanghai, China). After separation on a 12% acrylamide SDS-PAGE gel, the protein bands were transferred onto a PVDF membrane (Millipore, Massachusetts, USA). The membrane was incubated with different primary antibodies, against p38, p-p38, pERK1/2 and p-pERK1/2, respectively, which were purchased from cell signal Technology. A Bio-Rad ChemiDoc XRS+ image analyzer system was used to photograph the blots.

### Statistical analysis

All data were presented as the means ±standard error (SE), and the statistical analysis of the qRT-PCR assay was implemented using GraphPad Prism 8. The statistical significance of differences between JX and LW pigs was assessed using Student’s *t*-test, with *P* < 0.05 as the threshold.

## Supplementary Information

**Additional file 1.**

**Additional file 2.**

**Additional file 3.**

## Data Availability

The data sets supporting the results of this article are included within the manuscript and its additional files. The raw datasets generated in this study were deposited in the Sequence Read Archive under the accession number PRJNA631903.

## Abbreviations

SC: Subcutaneous
IM: Intramuscular
lncRNAs: long noncoding RNAs
JX pig: Jiaxing Black pig
LW pig: Large White pig
DEGs: Differentially expressed genes
DELs: Differentially expressed lncRNAs
GO: Gene Ontology
KEGG: Kyoto Encyclopedia of Genes and Genomes
MCC: Maximal Clique Centrality
MMPs: matrix metalloproteases
