# Supplementary material for: Comparative analysis of gene expression profiles in differentiated subcutaneous adipocytes between Jiaxing Black and Large White pigs
Source: BMC Genomics. 2021 Jan 19;22:61. doi: 10.1186/s12864-020-07361-9 (PMC7814706; doi:10.1186/s12864-020-07361-9)
Supplement: Supplementary file 1 — Additional file 1. [file 12864_2020_7361_MOESM1_ESM.pdf]

Supplementary Table 1 Summary of raw reads after quality control and mapping to the reference genome

Sample	Raw reads	Clean reads	Clean Read ratio	Total mapped reads	Uniquely mapped reads	Gene number (FPKM ≥ 1)
LW_SF1	163,015,236	152,234,246	93.39%	95.07%	90.22%	12,446
LW_SF2	165,689,668	152,444,042	92.01%	94.17%	87.20%	11,643
LW_SF3	166,398,866	152,161,886	91.44%	93.61%	86.05%	11,765
JX_SF1	161,091,938	152,250,878	94.51%	91.85%	84.79%	11,579
JX_SF2	159,503,542	151,692,558	95.10%	92.13%	85.59%	11,593
JX_SF3	162,238,544	151,565,872	93.42%	85.76%	92.91%	11,310

Supplementary Fig. 1 Characterization of subcutaneous adipocyte transcript in JX and LW pigs. A. Statistics of gene number according FPKM value among samples. B&C. Expression levels of mRNAs and lncRNAs indicated by log10(FPKM). D. Coding potential analysis of candidate lncRNAs was performed by using four conventional tools (CPC, txCdsPredict, CNCI and pfam). E. The exon number distribution of lncRNAs and mRNAs.

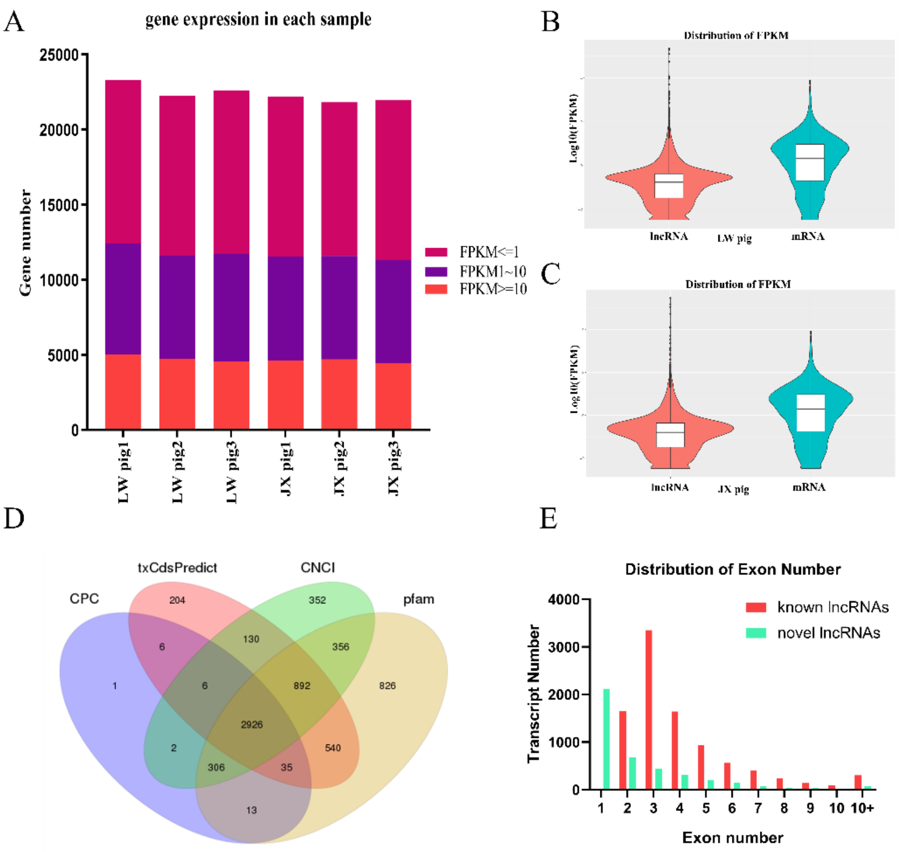

Supplementary Table 2 The top 50 expressed genes based on FPKM values of JX pigs

Gene Symbol	JX FPKM	LW FPKM
LGALS1	7773.65	5996.48
EEF1A1	7619.98	7326.90
RPLP1	6549.92	6488.11
COL1A2	4718.12	3841.24
TMSB10	4644.90	3826.81
RPS27	4523.23	3835.24
RPS19	4066.84	3764.01
RPS29	4003.07	3260.87
RPLP0	3943.62	3998.98
FTL	3832.95	5374.78
RPS12	3806.12	3966.53
S100A6	3637.09	2384.64
TMSB4X	3636.53	3053.08
RPS8	3292.06	2947.65
RPL35	2814.35	2665.29
SERF2	2701.96	2186.65
ACTA2	2617.83	2866.17
RPL37	2610.21	2327.09
VIM	2584.89	2895.58
CTSK	2492.33	1347.09
RPL23	2463.26	2098.41
RACK1	2461.45	2535.88
RPL26	2431.31	2471.48
GAPDH	2372.44	1881.21
RPLP2	2338.30	2351.18
RPS28	2299.64	2339.39
RPL17	2207.27	2018.07
TAGLN	2200.39	2563.98
RPS26	2179.53	2113.36
SPARC	2138.17	1846.41
RPL10A	2046.68	1866.33
RPS21	2011.14	2327.65
RPS15A	1973.47	1926.65
RPS18	1967.31	1847.04
RPS27A	1890.97	1752.33
RPS24	1853.98	1812.61
RPL29	1830.74	1703.81
RPS11	1806.13	1548.39
MYL6	1797.12	1745.54
RPL31	1694.36	1500.56

TPT1	1691.53	1711.40
LGMN	1687.39	1505.92
RPL19	1670.74	1663.57
RPL14	1668.24	1461.85
RPS7	1659.94	1549.12
RPL13A	1633.30	1450.34
RPL7A	1596.69	1705.34
RPS14	1585.58	1712.99
RPS23	1573.66	1499.43
RPL7	1550.16	1597.54

Supplementary Table 3 The top 25 up- and 25 down-regulated DEGs between the two breeds

Gene Symbol	JX FPKM	LW FPKM	Log2foldchange (JX/LW)	Up/Down	p-value
RPS17	123.4733	0	13.93041	Up	1.00E-300
LDHB	27.63	0.01	11.44782	Up	1.00E-300
PTCD1	1.05	0	10.33495	Up	3.2075E-101
TCEAL2	1.063333	0.03	5.191806	Up	4.04242E-47
SYNPR	8.793333	0.243333	5.167654	Up	1.00E-300
SLC7A3	1.006667	0.026667	5.137354	Up	3.9766E-99
CCT2	6.656667	0.21	4.984293	Up	1.00E-300
DDX24	1.69	0.1	4.086367	Up	5.9132E-192
C1QTNF3	25.73667	1.62	3.992309	Up	1.00E-300
HTR2A	1.466667	0.093333	3.718916	Up	1.276E-165
RAMP1	29.14667	2.566667	3.534632	Up	1.00E-300
AWN	7.866667	0.766667	3.37453	Up	4.0885E-167
CA4	5.216667	0.53	3.309405	Up	3.292E-191
H3-3A	8.103333	0.993333	3.153083	Up	4.4083E-102
CD207	2.556667	0.3	3.120928	Up	1.87208E-85
TLCD4	1.056667	0.126667	3.047772	Up	1.94E-227
CALCB	9.816667	0.783333	3.013125	Up	8.83E-185
FRMD7	2.306667	0.296667	2.952308	Up	3.1136E-186
FAM237A	4.18	0.736667	2.803847	Up	1.00E-300
CYP2J34	1.663333	0.24	2.802589	Up	1.05445E-92
IGFBP4	7.84	1.143333	2.782304	Up	1.1733E-155
MCHR1	2.68	0.403333	2.759758	Up	1.6859E-105
CHI3L2	4.853333	0.756667	2.694868	Up	1.9531E-236
CRSP-2	2.85	0.416667	2.640654	Up	5.43953E-47
MAB21L2	1.09	0.183333	2.572675	Up	9.66647E-84
SLA-DRA	0	5.22	-11.1169	Down	2.9928E-154
LGALS13	0	12.00333	-10.8663	Down	2.5382E-135
KRT5	0.036667	61.10667	-10.6362	Down	1.00E-300
GBP2	0	1.756667	-10.4668	Down	1.1703E-109
F13A1	0.006667	4.14	-9.24995	Down	1.00E-300

CCL24	0	1.023333	-9.02396	Down	2.06749E-50
LY86	0	1.72	-8.84923	Down	8.40197E-46
KRT8	0.013333	5.806667	-8.7705	Down	1.00E-300
UBC	1.89	408.48	-7.78886	Down	1.00E-300
KRT6A	0.073333	15.13	-7.65769	Down	1.00E-300
SLA-DRB1	0.036667	7.326667	-7.56995	Down	1.00E-300
COL17A1	0.03	4.716667	-7.27099	Down	1.00E-300
FAM199X	0.046667	4.146667	-6.46552	Down	1.00E-300
SPINK2	0.146667	13.27333	-6.42799	Down	1.2244E-222
SERPINB5	0.016667	1.153333	-6.38226	Down	4.0301E-138
TNFRSF1A	0.04	3.26	-6.28061	Down	2.1574E-281
TCEAL9	0.07	6.156667	-6.26086	Down	2.5601E-258
FEM1B	0.063333	4.51	-6.17989	Down	1.00E-300
MARCO	0.066667	4.646667	-6.05679	Down	1.2251E-299
SIGLEC1	0.023333	1.183333	-5.81012	Down	1.00E-300
SLA-DQA1	0.05	2.55	-5.68548	Down	2.5189E-135
TIMD4	0.033333	1.343333	-5.51382	Down	2.53182E-66
GRAP	0.046667	2.16	-5.4968	Down	7.0625E-172
PGM5	0.093333	3.87	-5.38985	Down	1.00E-300
TMSB15B	0.083333	3.463333	-5.38117	Down	7.4745E-90

Supplementary Table 4 The potential target genes of DELs

The table was provided in a XLSX file independently.

Supplementary Table 5 Sequences of Q-PCR primers for protein-coding genes and lncRNAs

Name	Sequence
ITGB3-RTF	GAAAAGGACTACCGCCCCTC
ITGB3-RTR	CACTCACAGTACTTGCCCGT
LRP10-RTF	ACCAAGACCGGATCCTTTTCC
LRP10-RTR	CCACAGGCCAGGTGTAGTTT
C1QTNF3-RTF	GAATACATGGAGGTGAGCGGA
C1QTNF3-RTR	TCCACAGTCCCAGTTCTAGC
LCN2-RTF	ATTGCCTCACCCTTCCTGGAT
LCN2-RTR	GGAGACTTAGGGCCATGGTTG
PLIN1-RTF	GTACAACCAGCTGCGCAAGAA
PLIN1-RTR	AGGTTCTTCTGGTTTGCAGGAG
PLIN4-RTF	ATAAGGCCAGGGAGCTGAGA
PLIN4-RTR	GGACCCAAAGAAGCTGCTCA
ISG15-RTF	GGTGAGGAACGACAAGGGTC
ISG15-RTR	GGCTTGAGGTCATACTCCCC
STEAP1-RTF	AGCAGTTTGGGCTTCTCAGT
STEAP1-RTR	GACCTGTTGATACGCCCAGT

ACKR3-RTF	GCTCTAAGGCAGTGATCGCA
ACKR3-RTR	GCTCCGAGTAGTCGAAGACG
KCNK12-RTF	CCGTAGTCCCCAGCAGATTC
KCNK12-RTR	AGATGGGGTTTAGTGGCAGC
MGP-RTF	TATCGCCACAGCAGAGATGG
MGP-RTR	TTGTAGGCGGCGTTGTATCC
REST-RTF	CAGAAGCAACTTGCACCACC
REST-RTR	TTGGTGTTTCCTGGCCACTT
LTCONS_00084841-RTF	AATCTCGTTCATCCATTC
LTCONS_00084841-RTR	ATGTCAAAGTGAAGAAATTC
LTCONS_00045964-RTF	CACAGCTTCATGCCCATTGT
LTCONS_00045964-RTR	GGGAGATTTAGGCTCCCGAT
LTCONS_00048531-RTF	AACCTCCCGCAATGTCTCAA
LTCONS_00048531-RTR	TTGTGTTCAGGTTGCGGTCT
LTCONS_00084076-RTF	GCTTACTCGGTGGCCATCG
LTCONS_00084076-RTR	CGAGTCAGAGGGAAACAAAGC
XR_002337668.1-RTF	CAGGTGCTTCTCTTGGGACG
XR_002337668.1-RTR	TGTGGGCCAGGTTCTTACAC
XR_002340445.1-RTF	GCCGCCAAGAAACGAAAACA
XR_002340445.1-RTR	GGGACCCTCAATGGTCTTGG
XR_001306655.2-RTF	TGCTGAGACGAGTGATGGCT
XR_001306655.2-RTR	ACAGGGCCACTCTCAAGTTT
XR_002346227.1-RTF	CCGGCCGCGGTATTTGA
XR_002346227.1-RTR	CTGGGACATCTGAGTTGCGG
XR_001299446.2-RTF	CAAATCCCGCAGTGTTCTCA
XR_001299446.2-RTR	TGGGGCAAGGCATCGAAAT
XR_303007.3-RTF	GGATCAGTGGGCTCAGTCTTT
XR_303007.3-RTR	GTGTTCATCAGACGATCCCCA
