# Supplementary material for: Comparative analysis of gene expression profiles in differentiated subcutaneous adipocytes between Jiaxing Black and Large White pigs
Source: BMC Genomics. 2021 Jan 19;22:61. doi: 10.1186/s12864-020-07361-9 (PMC7814706; doi:10.1186/s12864-020-07361-9)

Supplementary Fig 2: The original images of western blot in figure 6

A. Western blot analysis of protein lysates from SC fat tissue of two JX (JX 1 & JX2) and two LW pigs (LW1 & LW2). B. Western blot analysis of protein lysates from SC fat tissue of a JX (JX3) and a LW pigs (LW3).

A

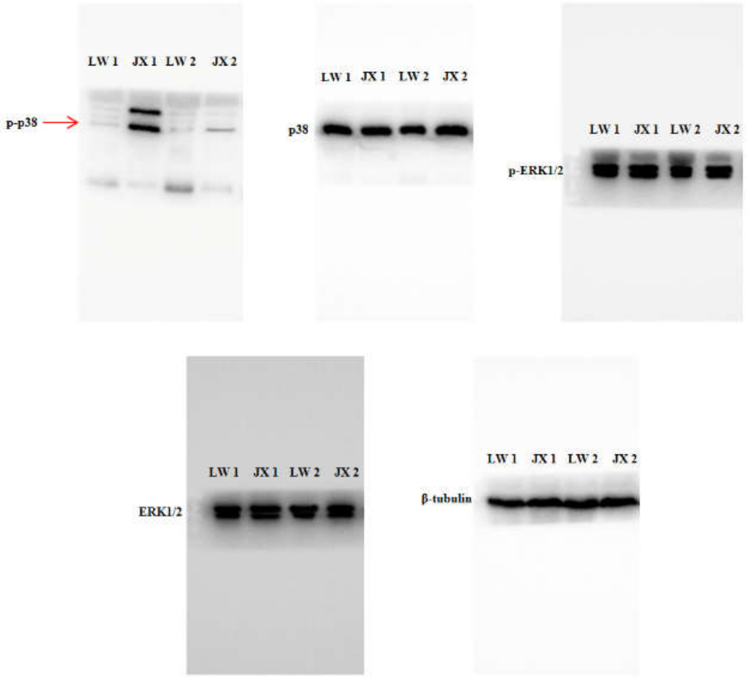

B

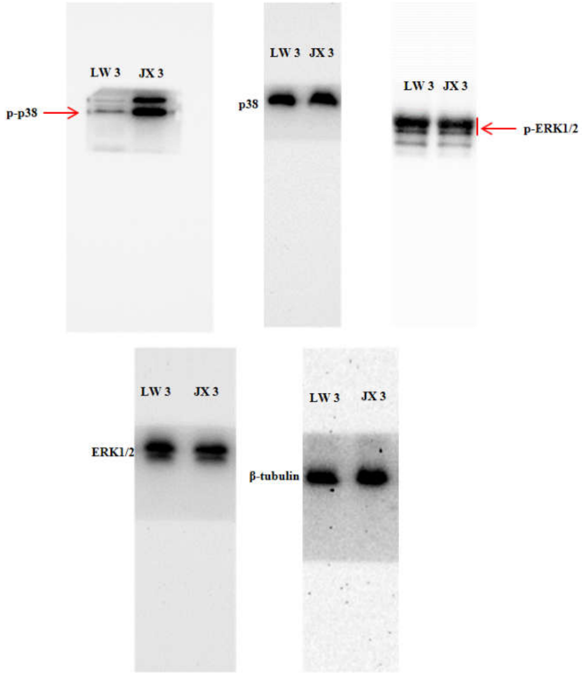

Supplement: Supplementary file 3 — Additional file 3. [file 12864_2020_7361_MOESM3_ESM.pdf]
